# H3Africa: a tipping point for a revolution in bioinformatics, genomics and health research in Africa

**DOI:** 10.1186/1751-0473-9-10

**Published:** 2014-05-08

**Authors:** Moses P Adoga, Segun A Fatumo, Simon M Agwale

**Affiliations:** 1 Computational and Evolutionary Biology/Bioinformatics, Faculty of Life Sciences, University of Manchester, Manchester, UK; 2 Microbiology Unit, Department of Biological Sciences, Nasarawa State University, Keffi, Nigeria; 3 Wellcome Trust Sanger Institute, Cambridge, UK; 4 Innovative Biotech USA Inc., Havre de Grace Corporate Center, Lab 1, 224 North Washington Street, Havre de Grace, MD 21078, USA; 5 Innovative Biotech, Keffi/Abuja, Nigeria; 6 International Health Research Group, Department of Public Health & Primary Care, University of Cambridge, Cambridge, UK; 7 H3ABioNet Node, National Biotechnology Development Agency, NABDA/FMST, Abuja, Nigeria

**Keywords:** H3Africa, Genomics, Bioinformatics, Genetics, Heredity, Health, Africa, NIH, Wellcome Trust

## Abstract

**Background:**

A multi-million dollar research initiative involving the National Institutes of Health (NIH), Wellcome Trust and African scientists has been launched. The initiative, referred to as H3Africa, is an acronym that stands for Human Heredity and Health in Africa. Here, we outline what this initiative is set to achieve and the latest commitments of the key players as at October 2013.

**Findings:**

The initiative has so far been awarded over $74 million in research grants. During the first set of awards announced in 2012, the NIH granted $5 million a year for a period of five years, while the Wellcome Trust doled out at least $12 million over the period to the research consortium. This was in addition to Wellcome Trust’s provision of administrative support, scientific consultation and advanced training, all in collaboration with the African Society for Human Genetics. In addition, during the second set of awards announced in October 2013, the NIH awarded to the laudable initiative 10 new grants of up to $17 million over the next four years.

**Conclusions:**

H3Africa is poised to transform the face of research in genomics, bioinformatics and health in Africa. The capacity of African scientists will be enhanced through training and the better research facilities that will be acquired. Research collaborations between Africa and the West will grow and all stakeholders, including the funding partners, African scientists, scientists across the globe, physicians and patients will be the eventual winners.

## Findings

The second set of grant recipients under a strategic partnership was announced in October 2013 [1]. Interestingly, this followed the announcement of first set of recipients the previous year [2]. Needless to say, this initiative can well be described as a tipping point for bioinformatics, genomics and health research revolution in this genetically diverse continent. Expectedly, this should excite all scientists, especially of African descent.

**Figure 1:**
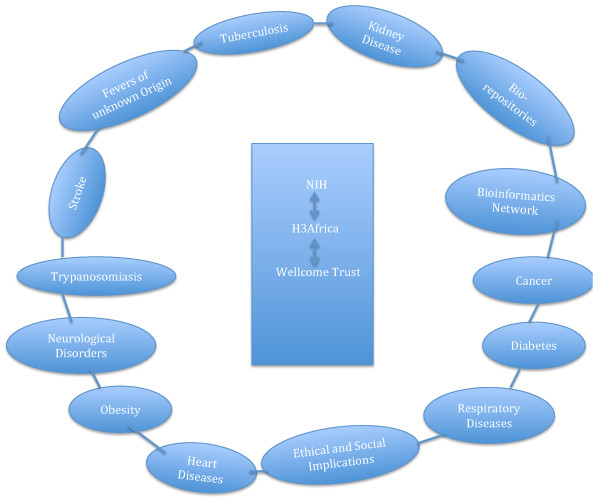
**Projects currently funded under the H3Africa initiative. **The NIH and Wellcome Trust are importantly at the centre of this initiative, with a resulting network of projects run by African scientists.

This potentially revolutionising initiative came into force in June 2010, when the NIH and Wellcome Trust announced a partnership that would see African scientists, in the first round of the award, utilize over $40 million in research grants over a period of five years [2]. The initiative, referred to as H3Africa, is an acronym that stands for Human Heredity and Health in Africa [1-4]. The projects currently funded under this initiative are demonstrated in Figure 1[1,2]. During the initial award in 2012, the NIH granted $5 million a year for a period of five years, while the Wellcome Trust doled out at least $12 million over the same period to the research consortium. This was in addition to Wellcome Trust’s provision of administrative support, scientific consultation and advanced training, all in collaboration with the African Society for Human Genetics [2].

Apparently pleased with the partnership, the NIH awarded to this laudable initiative 10 new grants of up to $17 million for another four years during the second round announced in October 2013. This brings the total amount so far awarded to the H3Africa to about $74 million [1]. This is by no funding standards a negligible amount. Therefore, such serious commitment from the funding partners in this mutually beneficial partnership deserves accolades not just from the African community of scientists and policy makers; but all scientists across the globe that stand to share in the many benefits this great initiative is poised to generate.

What this research consortium is set to achieve is aptly captured in the words of Dr Eric D. Green, director of the National Human Genome Research Institute (NHGRI), when he said; “These H3Africa awards demonstrate our continued commitment to furthering the capacity for genomics research on the African continent. Studying human diseases within populations with the greatest genetic variability and encouraging the contributions of our African colleagues should yield new insights about the role of genetics in health and disease.” NHGRI coordinates H3Africa in partnership with some NIH institutes and offices. It is interesting that the combined NIH and Wellcome Trust H3Africa initiative will now support research projects in 27 African countries and 93 collaborators across the continent [1].

Additional benefits of the programme are that it will build research capacity and collaborations in Africa. Some of the projects focus on developing a bioinformatics network and collection of specimens and data for storage in bio-repositories. This is critical for the future of genomics and personalized medicine [5]; and provides new opportunities for bioinformatics research in this continent. Moreover, the fact that resources from bio-repositories can be shared between laboratories both locally and internationally, demonstrates one more opportunity for local and international research collaborations inherent in this initiative.

Findings reveal that humans migrated out of Africa via Arabia thousands of years ago as part of general migration spreading across Europe, Asia and Australia [6,7]. Since this makes Africa the cradle of humanity, genetic diversity among humans decreases as distance from Africa increases. In other words, Africans are the most genetically diverse population. Therefore, searching for disease-causing genes requires the greatest number of markers among Africans [6]. This potentially provides opportunities for a better understanding of diseases with genetic pathologies. Regrettably, these opportunities have hitherto remained virtually untapped. Fortunately, the H3Africa initiative ignites hope, as it will enhance the capacity of African researchers for cutting-edge research, with a resulting better understanding of the environmental and genomic determinants of diseases. In the long run, this will improve clinical services and health outcomes in Africa.

Finally, with sustained commitment from all stakeholders, this partnership may be the long-awaited tipping point for a revolution in bioinformatics, genomics and health research in Africa with enormous benefits for the scientific community, physicians, patients, policy makers and all other stakeholders.

## Competing interests

The authors declare that they have no competing interests.

## Authors’ contributions

All authors contributed equally to this work. All authors read and approved the final manuscript.

## Authors’ information

MPA is the president, Regional Student Group (West Africa) of the Student Council of the International Society for Computational Biology (ISCB) and a faculty member at Nasarawa State University, Keffi, Nigeria. SAF is the pioneer president and founder of ISCB’s Regional Student Group Africa (2007–2009) and currently an H3AbioNet NABDA Node’s Visiting Research Fellow at the Wellcome Trust Sanger Institute and the Department of Public Health, University of Cambridge. He is the Vice-President of ASBCB (2011-present). SMA is the President/CEO, Innovative Biotech USA Inc and Innovative Biotech, Nigeria.
